# Medico-Chirurgical Transactions, Vol. XXI

**Published:** 1839-01-01

**Authors:** 


### MEDICO-CHIRURGICAL TRANSACTIONS. Published by the Royal Medico-Chirurgical Society of London. Volume the Twenty-First.

[Concluded.]

OF the twenty-four papers contained in this volume, twelve were fully noticed in our last number. The remainder will form the subject of the present article.

Two or three are of a miscellaneous character, and of small dimensions. We shall dispatch them first.

I. Note on the Comparative Prevalence of Calculous Diseases, &c. By Mr. Copland Hutchison, F.R.S. L. & E.

Our readers are probably aware that Mr. Copland Hutchison has endeavoured to establish two statistical facts of some interest and importance—one, that calculous diseases are comparatively unfrequent among sea-faring people; the other, that they are comparatively frequent in Scotland.

The former statement has met with some opposition. To this Mr. Hutchison alludes, and endeavours to meet the argument employed against him in the following manner.

"It has been stated, in opposition to my opinions, that although calculous diseases have been proved to be exceedingly rare among sea-faring people,—to amount, indeed, almost to a total exemption,—that such exemption arises more from the circumstance of this class of people having embraced their insular employment after the calculous diathesis is supposed to have been passed by—namely, the period of youth, than from any immunity they may possess from their particular situation and mode of life, according to my previous statements.

Those who have advanced this doctrine should, however, recollect that I have elsewhere distinctly observed, that boys, at the early ages of nine and ten years, were admitted into ships of war as midshipmen, officers' servants, or in the merchants' service as cabin boys; and it can be shewn, from data not to be disputed, that more than twice the number of operations for stone are performed on persons after the age of fourteen even, than before that period of life." 21.

Since Mr. Hutchison wrote his former paper, Dr. Henry Lee, of Charlotte Street, Bloomsbury, has pointed out to him a passage of Aretæus, which appears to lend some gentle confirmation to his views. Speaking of the *cure* of calculous diseases, Aretæus says:—"but diet and anointing, and sailing and passing one's life at sea—all these are remedial in diseases of the kidneys."

Mr. Hutchison adds;—

"I have applied to Sir William Burnett, the Physician-General to the Navy, for an account of such cases of stone and gravel as may have been admitted into the naval hospitals at home and abroad, from April 1830, the period of my last communication on this subject to the Society, up to the present date, and the answer returned is as follows.—'I have caused the returns of the naval hospitals at home and abroad to be carefully examined from April 1830 to the present time, with reference to the prevalence of calculous disorders, and the only instance of the kind is one case of renal calculi, in Malta hospital, in Michaelmas quarter, 1833; the patient was purser of the Pelican sloop of war.' P.S. 'He was discharged, cured, in the same quarter.'

'Signed, William Burnett,

'26th Nov. 1836.'

The average number of seaman and marines annually voted by parliament from 1830 to the present date, *including* 2,000 *boys*, has been 30,000. The latter are particularly specified, in the navy estimates, as boys." 23.

Such is the amount of confirmation, afforded to his former statements, in Mr. Hutchison's present paper. We would merely suggest for Mr. Hutchison's consideration, and as reasonble elements in his calculations, that stone is, on the whole, the disease of early and advanced life—that a delicate boy would not go to sea, this stormy element being usually selected by the hardy and the wild—and that the old sailor will frequently have quitted the navy and its institutions, when calculous disease has overtaken him. We do not say that these considerations explain all the immunity from stone on the part of sailors, contended for by Mr. Hutchison. But we think he will admit that they deserve to be taken into the account.

II. HISTORY OF A FEMALE WHO HAS FOUR MAMMÆ AND NIPPLES. BY ROBERT LEE, M.D. F.R.S., Physician to the British Lying-In Hospital, and Lecturer on Midwifery at St. George's Hospital.

Our friend Dr. Lee is never idle. Ever on the watch for some interesting fact, or some broad generalization in the field of science, his own department of it is continually receiving some accessions at his hands. The following fact is curious—

Mrs. ——, æt. 35, was delivered prematurely of a still-born child on the 21st July, 1835. Soon afterwards, the mammæ became excessively painful and distended, and she had a severe attack of fever with delirium. Though the symptoms became daily more aggravated, a week elapsed before she would permit the condition of the breasts to be ascertained. On inquiring into the cause of this unwillingness to allow the necessary examination of the mammæ to be made, Dr. Lee was informed by her sister-in-law, that she had two mammæ and two nipples on each side, and that this peculiarity, which she was anxious to conceal, had been observed ten years before, when her first confinement took place.

After long entreaty, Dr. Lee obtained leave to inspect the breasts, and was surprised to find that there were two on each side, as had been represented; the two of the same side being separated by a deep oblique depression. The inferior or pectoral mammæ, as they were afterwards termed by Sir Astley Cooper, were fully developed and in their natural situation, and their nipples, areolæ and glands, presented nothing unusual in their appearance. Near the anterior margin of the axilla, a little higher up on each side, was situated another mamma, about one-sixth the size of the others. The nipples of these were small and flat, but when gently pressed, a milky fluid, which had all the external characters of the milk secreted by the other breasts, flowed copiously and readily from several ducts which opened on their extremities. When milk was drawn from the lower breasts, a small quantity usually escaped from the nipples of the superior breasts, and when the draught came into the former, the latter invariably became hard and distended.

Mrs. —— had previously borne several living children, and five years before this period had twins, when she had a severe attack of uterine inflammation, and suffered much from painful distention of the two upper breasts. In consequence of the flatness of their nipples, she has never been able to suckle any of her children with these. The vagina, orifice of the uterus, and all the other organs, besides the mammæ, in this female, are well formed.

"I mentioned," continues our excellent friend, "this case to Sir Astley Cooper at the time it first came under my observation, but he did not see it with me until the 28th February, 1836, several months after the secretion of milk had entirely ceased. When Sir Astley saw the mammæ, he said there could be no doubt that there were two on each side, an axillary and pectoral breast, and that nature had separated them completely from each other. He considered it proper that some record should be given of a case, which he thought to be without a parallel in this country.

Mrs. —— again became pregnant, and was safely delivered on the 19th July, 1837, of a living child, which she now suckles with the pectoral breasts, and the axillary breasts again present the same appearances as those which have now been described.

The preceding case furnishes one of the best examples of quadruple mammæ in the human subject which has yet occurred." 268.

Dr. Lee has collected the particulars of five other cases from foreign authors. The knowledge of the occasional occurrence of the fact may possibly be useful. At all events, the fact itself is one of the "curiosities" of Nature. Dr. Lee adds in conclusion :—

In some women only one breast has been developed, others have had two nipples placed on one mamma, and a few individuals have had three breasts, two in the natural situation, and a third situated in the middle of the two others. Only one case has been recorded of five mammæ in the human subject.*

III. Results of Poisoning by Sulphuric Acid. By John Wilson, M.D. Physician to the Middlesex Hospital.

Two cases are detailed.

1. In one, the patient lived for six months after swallowing a part of two-pennyworth of oil of vitriol. She ejected, in a violent fit of coughing, a cylindrical tube, about eight or nine inches in length. On the morning of the 14th of November, she had a shivering fit, preceded by vomiting, and succeeded by great pain in the region of the stomach, &c. and fatal collapse. She died on the 17th.

*Examination*, eighteen hours after death.—Body extremely emaciated; the lower two-thirds of the œsophagus thickened and narrowed, internally very vascular, irregular, and softened; the upper third shining like an old cicatrix. In the stomach, opposite to the spleen, was an opening of the size of a half-crown piece with softened edges; there was great softening of the mucous membrane of the stomach; the abdomen contained a quantity of dark-coloured fluid, but no peritoneal inflammation.

In the interval between her swallowing the acid, and her fatal seizure, she had suffered, amongst other symptoms, considerable difficulty in swallowing. Some thought it would be advisable to pass a bougie down the œsophagus. But Sir Charles Bell and Dr. Wilson thought otherwise, and the disssection shewed that they were right.

2. In the second case, death occurred twenty-two hours after swallowing from two to three ounces of strong sulphuric acid, which remained on the stomach for a quarter of an hour.

*Inspection*, fourteen hours after death.—Lining membrane of the mouth,

* Dict. des Scien. Med. Tom. XXXIV. p. 529.

pharynx, and œsophagus, of a silvery grey speckled appearance, like a snake's skin, from some of the carbonized matter adhering to the deeper parts of the rugæ, the more prominent being of a lighter appearance; the membrane of the tongue easily peeled off.

The stomach was very much distended, but contained only air, and when opened, its mucous coat was nowhere visible, from its entire surface being covered with a black pitch-like substance, which did not wash off with ease, and when scraped it shewed the mucous coat of a pink colour, much swollen, but entire: the commencement of the duodenum had a similar appearance, which soon became less marked, and nearly disappeared at the beginning of the jejunum, when it gradually assumed the greyish appearance, somewhat like that of the œsophagus, but of a much more dull colour, all of which seemed to terminate with the ileum.

The peritoneaal coat of the small intestines, and particularly that of the stomach, was much inflamed, but no albuminous deposits were seen.

### IV. History of a Case of Popliteal Aneurysm. With Observations. By Samuel Hadwen, House-Surgeon to the Lincoln Hospital.

This case is interesting on several accounts, though it does not seem to us to support altogether the observations appended to it.

*Case.* John Asman, aged 23, a muscular man who had been accustomed to violent exertions, was seized with pain in the right leg and knee, succeeded by swelling. The complaint was thought to be rheumatic.

Three months afterwards, July 16th, 1837, he came under the care of Mr. Hewson, surgeon to the Lincoln Hospital. He had then an aneurysmal tumor in the popliteal space, about as large as an orange, with diffused tumefaction around it. There was distinct pulsation, synchronous with the heart, and clear bruit in the swelling, especially at the inner side of the calf. Pressure upon the femoral artery suspended the pulsation, rendered the tumor soft, and diminished its size. The heart and arterial system, examined with the stethoscope, appeared perfectly healthy.

On the 18th, Mr. Hewson placed a ligature upon the superficial femoral artery at the margin of the sartorius muscle. Before the patient was removed from the table, it was observed that the aneurysmal tumor was as large as ever, and the tension greater than it was before the application of the ligature; there was, however, no return of pulsation. He suffered very severe pain, for a few seconds after the ligature was tightened, down the leg, and a slight irregularity of the pulse, not amounting to an intermission, could be occasionally but unfrequently perceived.

An hour after the operation, no pulsation could be perceived in the tumor; but after three hours more it had returned with some force. Twelve hours after the operation the tumor did not appear quite so large as it had been, and was of the natural temperature; it was, however, deemed expedient to envelop it in flannel. There was a regular, but indistinct pulsation in the tumor, which was not synchronous with the pulse.

19th. Integument over the tumor yellow, tense, and resistant. A feeble pulsation could be felt, and an obscure murmur heard.

27th. Pulsation not perceptible in femoral artery below the ligature, but distinct in the tumor at the ham, which was still hard and resisting. Leg, from knee to toes, beginning to assume an œdematous character, and of great size. Wound healed, save where the ligature projected through it. Heat of leg great. Occasional paroxysms of severe pain, extending from the ham down the leg.

29th. Temperature between the toes of each foot 101° Fahr.; at the external surface of the calf, which was in contact with the bed, 102°; the same part of the unaffected leg, 98°. On applying the fingers to the middle of the thigh there was felt a pulsation, and, with the stethoscope, there could be traced a distinct arterial bruit along the inner side of the thigh in the course of the artery, from about three inches above the knee to the groin, least audible at the lower part, and gradually becoming more loud to a little above the ligature, where it was very strong and distinctly heard. Next day it was found that pressure on the artery at the groin completely suspended the pulsation in the sac, which was not the effect of pressure about the middle of the thigh, where the bruit was heard.

About 10, p. m. of that day (30th) about eight ounces of florid blood issued from the wound. There was some puffiness around the cicatrix, and about an inch and a half to the outer side, a distinct pulsation. A little pressure and cold were applied.

"31st. At five o'clock this morning there was a slight escape of blood, not more than an ounce. About twelve at noon the hemorrhage returned with such violence that an arterial jet was forced out between the dressings to some distance. It was decided that the femoral artery should be tied again immediately below Poupart's ligament, which was accordingly done by Mr. Hewson.

The ligature was firmly tied, and the beating previously observable at the seat of the former ligature, and in the surrounding tumefaction of effused blood, immediately ceased. It was also thought the aneurismal sac and calf were less distended; the pulsation in the former, and the bruit along the course of the femoral artery, were stopped.

In the evening it was found that there had been a slight oozing of blood from the situation of the first ligature, and in the surrounding tumefaction, which was reduced in size, a decided pulsation could be felt, and, with the stethoscope, a faint bruit." 323.

The tumor was softer; temperature between the toes 90°, gradually rising as the instrument advanced up the leg, and at the calf the mercury stood at 102°.

On the 1st August, a bruit was no longer perceptible with the stethoscope. At 8, p. m. of the 2nd, the bleeding suddenly recurred from the wound in the thigh where the first ligature had been applied. Pressure immediately above the wound controlled it, but, as soon as it was removed, the blood, in a stream as large as a quill, was projected upwards a foot and a half. The cicatrix was laid open, and an ineffectual attempt having been made to find the mouth of the bleeding vessel, amputation of the limb was immediately performed. The upper part of the artery was found to be quite separated from the lower, upon which the ligature still remained, and to present an open mouth.

Great collapse was the immediate effect of the operation, but he rallied from this, and went on well enough until the 21st. At 4, a. m. of that day the wound at the groin was observed to bleed; the blood was florid and arterial, and did not come away in a full stream, but gently oozed up, and apparently did not amount to more than five or six ounces. A compress of lint and adhesive plaster was applied. Digitalis was given, and his generous diet gave way to one of milk.

At 11, a. m., there was a return of hæmorrhage to a greater extent. The pulse was full and bounding. It was proposed to tie the external iliac artery, and secure the epigastric and circumflexa ilii; but the patient would not consent. He was therefore bled in the arm till the pulse became smaller and weaker, and a bladder containing salt and ice, pounded together, was applied over the groin.

About six o'clock in the evening a slight discharge of blood took place, and later at night the pulse rose in volume and strength. The blood removed in the morning presented a thin buffy coat.

22d. A very large and alarming bleeding suddenly burst forth at six o'clock, p. m., which produced a frightful effect, and placed the poor fellow in greater jeopardy than he had ever apparently been before. At his request, the operation was therefore instantly performed; brandy having been first given in small but frequently repeated doses.

The mode adopted by Sir A. Cooper was followed. When the peritoneum was arrived at, the epigastric artery was nearly exposed at the lower part of the wound, and by a little careful dissection, was cleared and tied, the subjacent membrane having sustained no injury. The peritoneum was then drawn to the inner side of the wound along with the cord, and the external iliac having been brought plainly into view, an armed needle was passed beneath it, and the ligature securely tied. The pulsation at the groin ceased. He nearly sank during the operation, but, under the influence of stimulants, he pulled through it. He did well until the 28th, when, at noon, hæmorrhage again appeared at the wound in the groin, and it is thought many ounces of blood were lost. It flowed at first gently from the part, afterwards in a larger stream, but not in a jet. Pressure with the hand restrained it. Graduated compresses of lint were carefully applied, and a truss so adjusted as to bring the pad to make firm pressure directly upon them. The truss, from the tightness with which it was applied, produced pain, which, however, an anodyne relieved. On the 30th, the truss was removed, the wound found to be healthy, and the instrument re-applied. Pulsation of the internal pudic artery was distinguishable. On the 4th of September, the stump was healed. On the 29th day, the ligature came away from the external iliac. The wound cicatrized, and, on the 101st day after the superficial femoral artery was tied, the man was down stairs, and fast recovering flesh and strength.

The observations of Mr. Hadwen are brief. He points out, what has often been insisted on, the disadvantages to which ligature of the common femoral artery is exposed, from the variable point of origin of the profunda and internal circumflex, as well as from the contiguous origins of the epigastric and the circumflex iliac branches.

"We are acquainted," he goes on to state, "with six recorded instances in which the common femoral artery has been selected for the application of a ligature, and two not hitherto given to the public. The dangerous hemorrhage which led the talented Abernethy to the performance of an operation never before attempted, was produced by a ligature placed upon this vessel. Sir A. Cooper has twice tied this artery; in one instance with success, in the other, hemorrhage arose on the fourteenth day, and death was the consequence. Sir B. Brodie has also tied this artery, and the result was hemorrhage and death. Dr. Murray applied a ligature to it, and owing to a violent bleeding which placed his patient, when the ligature was about to separate, in the utmost danger, he very properly tied the external iliac. Mr. Ivory tied this vessel, and in consequence of subsequent bleeding was under the necessity of taking up the external iliac. These are the six cases already recorded. In five of them violent bleedings followed, and in two of them death was the consequence, and would, in all probability, have occurred in the remainder had not the external iliac been tied. The two other instances alluded to occurred to Mr. Hewson. One was attended with a favourable result, the other with such bleeding that nothing, probably, but the operation to which it led, could have prevented a fatal termination. So that of eight cases in which a ligature was applied to this artery, six were attended with consecutive hemorrhage, two with death, and two with a favourable separation of the ligature; giving to this operation a highly dangerous character.

Contrast with this the result of tying the external iliac. Mr. Hodgson, when he published his work on the arteries, knew of twenty-two cases where the iliac was tied, and not in one of these was there any secondary hemorrhage. Since that period the operation has been performed a great number of times, and, as far as I can ascertain, with the same exemption from this alarming occurrence. I cannot, indeed, find a single case recorded of bleeding attending the separation of a ligature placed upon this artery; so that it may be said, not merely, as Mr. Hodgson observes, that the external iliac may be tied with as much safety as any artery to which a ligature has been applied, but that, of all the large vessels of the human body, it is the one that may be tied with the greatest security, as far as the effects of the operation are concerned, and with the best effects upon diseases to which it is applicable.

There is no case, except that of wound of the artery at the groin, in which tying the common femoral possesses any advantage over the ligature of the iliac; I am therefore justified in concluding that the common femoral artery ought never to be selected, in any case of disease, for the application of the ligature, and that the operation should be exploded." 330.

We fully agree with Mr. Hadwen, indeed similar sentiments have been more than once expressed in this Journal, on the dangers of tying the common femoral artery. But there are two points, one of fact and one of doctrine, on which we cannot go so far as he does.

The point of fact is this. Mr. Hadwen says there is no authentic instance of secondary hæmorrhage after ligature of the external iliac artery. Singularly enough, his own case *is* such an instance. The patient recovered it is true, but so fortunate a result cannot always be counted on. We believe there are *two other* instances on record. In a case in which Sir Astley Cooper tied the external iliac, fatal secondary hæmorrhage occurred, from the site, as we have understood, of the epigastric artery. And a similar accident happened to Dupuytren. We remember reading the latter case, and, if we are not much mistaken, it was copied into an early number of this Journal.

Here then are three facts contrary to the supposition that secondary hæmorrhage has not followed the ligature of the external iliac artery.

The point of doctrine hinges on the point of fact. If hæmorrhage has followed ligature of the external iliac, it follows, of course, that this operation is not one of such absolute safety as is represented. Now the operation on the lower part of the vessel is open to strong physiological objections. The ligature is applied, by the method of Sir Astley Cooper, close to the origin of the epigastric and circumflexa ilii. There is insufficient room for the inferior clot, and the very reason which makes the ligature of the common femoral dangerous, makes (not quite to the same degree) the ligature of the lower part of the external iliac dangerous too. This consideration, as well as some others connected with the method of proceeding, have almost proscribed the operation of Sir Astley Cooper from practice. It is little more than what the French call a "Procés d'Amphithéatre."

Before we conclude, we would remark that the idea of the great safety of ligature of the external iliac, in any part of its course, is exaggerated. Of three cases of this operation, which have occurred under our immediate observation, two have been fatal, and one (the high operation) was attended with secondary hæmorrhage. At the time when Mr. Hodgson's statistical calculations were made, there had been a run of luck in favour of the operation. This has often happened with lithotomy.

### V. Account of a Case of enormous Ventral Aneurysm; with the Post-mortem Appearances. By Sir David J. H. Dickson, M.D. F.R.S. Ed. &c. &c. &c. Physician to the Royal Naval Hospital, Plymouth.

Our friend Sir David Dickson is one of those who do not slumber at their post. The facts which occur at the Naval Hospital are sure, if valuable, to be communicated to the profession.

*Case.* A gunner, aged 36, was sent to the hospital at Jamaica, for reputed paraplegia, on the 22d September, 1836; discharged invalided on the 16th December; and received into the Plymouth Hospital on the 20th of March, 1837.

On admission, he complained, chiefly, of pain and uneasy feelings in the sacral region and loins, attended with weakness, partial loss of power, and numbness in the lower extremities, and imperfect command of the sphincter muscles; but his general health was not materially impaired. There was also a deep-seated and ill-defined hardness, or swelling, in the left side of the abdomen, which was at first referred to an affection of the spleen, but which, on further examination, was discovered to be a large diffused pulsating tumor, either in contact with the abdominal aorta, or more probably arising from an aneurysm of that great trunk itself or the common iliac artery: and thus the deep-seated pains, and numbness in the sacral region and thighs, at first simulating rheumatism, and afterwards lumbar abscess, as well as the occasional alternations of loose and torpid bowels, eneuresis, &c., were accounted for, by the compression of the vessels and nerves, and especially of the hypogastric plexus. Although the tumor enlarged, his general health, on the whole, improved, and on the 6th of September he was as well as usual. But on that day, soon after ascending some stairs, he was seized with excruciating pain in the right iliac region, followed by excessive faintness, and a death-like paleness of the countenance, indicating the rupture of the aneurysm, and, after suffering much pain, he expired at 6, p. m.

*Dissection.* "Upon opening the cavity of the abdomen, a small quantity of bloody serum escaped. The posterior reflection of the peritoneum, on the right side, presented an ecchymosed appearance, from subjacent semicoagulated blood, which, effused in vast quantity, had raised the membrane from its attachments behind, and separated the laminæ of its different processes from each other. The blood was discovered to have escaped by an ulcerated opening of the size of a shilling, in the side of an immense tumour near to the right kidney, which it had displaced forward and laterally; and which, on further examination, proved to be an enormous aneurysm of the descending aorta. The aneurysmal dilatation, upon further investigation, was found to commence from the posterior part of the artery, two inches above the cœliac axis, by a kind of neck, which extended to two inches and a half above its division into the iliac trunks; where, suddenly bulging out, it expanded over the whole of the abdomen. The tumour was so immense indeed, that with the exception of the cæcal region, from which it diverged to the left, it might be said to occupy the epigastric, both hypochondriac, the umbilical, and left iliac regions, and the pelvis. But to describe it more minutely, the aneurysm, accommodating itself to the concavity of the diaphragm, to which, as well as to the posterior inferior surface of the liver, it intimately adhered, lay behind the hepatic vessels and ducts, the pancreas, duodenum, &c. It was attached to the false ribs and spine, and descending between the latter and the vena cava and aorta, it continued downwards behind the ureters and iliac vessels, but separated from them by the iliac fascia, which, greatly condensed, formed one of its anterior coverings, and beneath which it insinuated itself. The tumour thence protruded in a conical form under Poupart's ligament, and appeared like an aneurysm of the left iliac artery. This vessel lay in front and the ureter crossed it obliquely, while the psoas lay internally. The iliac muscles and crural nerve externally, and the great sciatic nerve were closely attached to its posterior inferior part. When this immense aneurysm was laid open, it was found to be nearly filled with coagulated blood, of the consistence of wet clay, and some concentric layers of nearly colourless fibrine adhered, though not vascularly, to its walls. The lining of the sac, on the tumour being emptied, appeared of a vivid red colour, mottled with osseous scales, deposited in the fibrous tunic, which, in a great measure, prevented its collapse. A careful examination was then made of the coats of the aneurism: the external covered it completely, except where it adhered to the spine, where the tunics had entirely disappeared, and the last dorsal and first lumbar vertebræ were also partially absorbed. The middle coat was continued over the sac, or so gradually lost in the other coverings, which in some places were increased in thickness to nearly two inches, that its termination could not be detected. The internal tunic was continued for some way into the sac, where it became broken down, and undistinguishable from the adjoining clots. The abundant deposition of ossified matter in the middle coat prevented the collapse of the artery, from the pressure before and behind; and, by maintaining its cylindrical form, preserved a channel for the blood. Two small appendages, resembling knuckles, of intestine, were observed on the iliac portion of the great tumour, and containing blood of the same appearance; but they were distinct from it, being closed by the adhesion of their necks; and their walls were thin and of a purple grape colour. The abdominal and thoracic viscera, generally, were normal, with the exception of some pleural adhesions; and the body was muscular and not much emaciated. So intimate was the attachment of the tumour to the spine, that the lumbar and three dorsal vertebræ were removed with it." 405.

A remarkable instance of aneurysm!

The next two articles are of a physiological character.

VI. ON NECROSIS; BEING AN EXPERIMENTAL INQUIRY INTO THE AGENCY ASCRIBED TO THE ABSORBENTS, IN THE REMOVAL OF THE SEQUESTRUM. By GEORGE GULLIVER, Esq. Assistant Surgeon, Royal Horse Guards.

The object of Mr. Gulliver has been to determine, by experiments on dogs and rabbits, in the first place, what becomes of the dead bone in necrosis; and, in the second place, the means by which it is replaced. But the present paper is exclusively devoted to the examination of the first question—whether dead bone admits of removal by absorption.

"While engaged in the formation of the catalogue of the museum of the Army Medical Department at Chatham, 1829, I was led, from the examination of numerous specimens of necrosis in that collection, to entertain a suspicion that the doctrine of the absorption of dead bone, so confidently asserted in the schools as an ascertained fact, might notwithstanding be founded in error,—and a further attention to the subject tended to confirm this persuasion. As far as I could judge from my own observations, it did not appear necessary to attribute the form and appearance of the dead bone to the agency of the absorbents after it had ceased to be a part of the living body, the facts appearing susceptible of explanation otherwise; while many cases presented phenomena altogether at variance with the received opinion." 3.

Mr. Gulliver observes that the facts which are brought forward in proof of the absorption of dead bone are—the gradual disappearance of the sequestrum in many cases of alleged necrosis; the irregular and eroded state of the dead portion; the contact of granulations with the indentations on its surface; the absorption of the fang of a transplanted tooth; and finally, (on the authority either of Mr. Abernethy or of Sir William Blizard, that portions of dead bone have diminished in weight, after having been kept in contact with the granulations of an ulcer.

1. Upon the latter fact Mr. Gulliver remarks, in a note—

"In Mr. Palmer's edition of the works of John Hunter, the following note appears. 'Portions of dead bone were often observed to be entirely absorbed in cases of necrosis; and in some experiments made by Mr. Thomas Blizard, in which disks of bone were bound on over ulcers, the surfaces of these disks were found to be eaten out, or destroyed, just as in common caries.' Vol. I. p. 255. The result of my experiments justifies the belief that there must be some mistake in this statement." 4.

And he mentions a suggestion of Dr. Davy, that, if dead bone be subjected to the combined action of air, heat, and moisture, it might lose weight from the decomposition of its animal part, especially if the discharge were long confined.

2. In opposition to the reputed fact of the absorption of dead bone confined in old, Mr. Gulliver urges that Wiedmann, F. Ribes, Jules Cloquet, had each observed examples in which it had been incarcerated for years, without apparent diminution, in a new osseous cylinder, from the internal surface of which more or less purulent matter was secreted. Mr. Liston adduces cases of detached pieces of bone in similar circumstances long remaining unaltered in form, in some of which amputation of the limb was required from the irritation of a dead portion so small, that it is inconceivable how it could have resisted absorption, if that were the process employed by nature for the removal of dead bone; and Mr. Syme mentions similar instances.

We would remark that a number of negative instances do not disprove the occasional occurrence of a fact. No one contends that a sequestrum is always absorbed. Mr. Gulliver goes on to remark:—

"But if the sequestrum is not absorbed, what becomes of it? It may be remarked, in the first place, that they are not all cases of necrosis that have been so denominated. Under this head, in the museums of anatomy, a class of specimens is sometimes presented to our notice which seem to me to admit of an explanation differing from that commonly assigned to them. These are generally the shafts of the long bones, prodigiously thickened and irregularly perforated with holes for the transmission of blood vessels, or by cloacæ leading to the cavities of abscesses, and sometimes singularly crooked and mis-shapen, as if at one period of the disease they had been softened, and influenced by mechanical force. In the centre of such bones a very small portion is sometimes found dead and detached, but more frequently the shaft is simply very thick and dense throughout. The former have frequently been regarded as examples in which the absorption is nearly effected; the latter as the completion of this process. It is probable that both are instances of long continued inflammation of bone, the first attended with death and separation of a small central fragment, which had afterwards undergone no alteration of form, and that the second was never at any period a case of necrosis.

The deposition of a cylinder of new bone around the old one, is not an absolute proof of the death of the latter, as I have had frequent opportunities of ascertaining in the course of my experiments. Nature often exhibits a prospective contrivance in the formation of a new osseous shell, or in the enlargement of a part of the old shaft, before actual necrosis has taken place; a fact which has not escaped the observation of Mr. Russell and Dr. Macartney. In the museum of St. Bartholomew's hospital, there is the tibia of a dog incased in a shell of new bone, and partly detached, but the injection has run pretty freely into the old bone.* In such instances, the part which has suffered the most intense inflammation may become partially eroded, and gradually removed by absorption, if it retain its vitality long enough, while a deposition of new osseous matter gradually supplies the loss, death of the old bone having formed no part of the phenomena. This is probably the explanation of many cases of alleged absorption of dead bone. But if a piece of bone truly dead be inclosed within a new osseous cylinder, then it is indeed a bad case of necrosis, which the patient will carry to the grave with him, unless relieved of the sequestrum otherwise than by absorption.

The worm-eaten appearance on the surface of many sequestra may be explicable in two ways. The most numerous examples of this kind are those of necrosis of the inner layer of the shaft of the long bones, with thickening of the outer portions,—a form of disease known to Bordenave, Haller, Collison, and Tenon, and since more fully explained by Brun, Brugnoni, Penchianati, Dr. Knox, Mr. Syme, and others. In such cases, irregular death, and separation of a portion of a bone, may be expected to produce an equally irregular surface: the part would not necessarily die in a determinate form, any more than in cases of sloughing of soft textures; and when the outer layer of an entire cylinder of necrosed bone presents erosions on its surface, it seems more reasonable to refer these to the effect of the ulcerative process, while the part retained its vitality, than to the action of the absorbents after its death.

---

* It is proper to notice that Mr. Stanley considers this to be doubtful. The preparation will be found under the head of "Bone," No. 10.

The aspect and situation of the granulations is equally inconclusive. They are seen to be extremely vascular, and accurately corresponding to the indentations on the under surface of a superficial layer of dead bone in progress of exfoliation, a case in which it has not often been supposed that the dead portion suffers diminution from the absorbents, the action of which is confined to the surface of the living bone in immediate contact with that about to be separated. The vascular structure adjusted to the superficial excavations on the surface of the sequestrum, is what might be expected from the work of exfoliation in some instances, or from the extension of the ossific process into the vacant spaces in others." 7.

3. Mr. Gulliver is not aware that the absorption of the fang of a transplanted tooth is a well-authenticated fact; but, if so, it would seem to indicate that the tooth, having preserved its vitality, had become a part of the living body to which it was attached, and accordingly subject to its laws.

Such are the reasonings of Mr. Gulliver. It will at once be admitted that they are fair and forcible, that they explain many supposed instances of absorption of dead bone, and that they tend to throw doubt upon the doctrine which unreservedly avails itself of such a process. But they are certainly not so staggering nor so conclusive as to make us deny its existence.

We turn to Mr. Gulliver's experiments, nineteen in number. We shall select such as are calculated to establish leading points.

*Experiment* 1.—A thin portion of the surface of the shaft of a human tibia was kept in contact for seventeen days with a large ulcer, studded with granulations, in a man's leg. The bone having been removed, dried, and weighed, was found to have undergone no alteration either in weight or appearance.

In the next three experiments a portion of human bone was introduced into a seton in the back of the neck. The following may be considered a representative of all.

*Experiment* 4.—A section of the shaft of the human humerus, weighing 10·7 grains, and comprehending the entire thickness of the bone, was introduced into a seton at the back of a man's neck, and retained there sixty-five days. The suppuration was at first scanty, but became copious during the latter five weeks. The bone was removed, and found to have undergone no alteration in appearance, but it had increased exactly one-tenth of a grain in weight, probably from some albuminous matter which was not entirely dissipated by drying.

In the next four experiments, a portion of bone, in three instances human, was introduced into the soft parts of a dog's leg. Two of the experiments will exhibit the results of the four.

*Experiment* 6.—A portion of the shaft of a dog's thigh-bone, weighing 7·8 grains, was introduced deeply between the muscles and periosteum of another dog's leg, and kept there two months. Suppuration was soon established, and continued till the animal was killed. The bone had suffered no alteration whatever. The cavity in which it had lain was very vascular, being made deeply red by injection with size and vermillion.

*Experiment* 8.—A thin portion of the shaft of the human humerus was placed in the subcutaneous cellular tissue of a dog's leg, and allowed to remain there four months. The wound soon healed, and continued sound till the animal was killed. The bone had suffered no change whatever: it adhered slightly to the cellular substance, so as to stretch out the filaments of the latter as the bone was pulled away.

In the remaining eleven experiments, portions of bone, either human or otherwise, were introduced into the medullary canal of the tibia of rabbits, and retained there for a longer or a shorter time. The main experiments are:—

*Experiment* 11.—A portion of the shaft of a rabbit's tibia, weighing 2·1 grains, was put into the medullary canal of the tibia of another rabbit, and retained there thirty-four days.

The foreign bone was found to have undergone no change; it was surrounded by highly vascular lymph, and there was a large cyst, which had not yet burst, containing a white, concrete, purulent matter, and communicating with the cavity of the tibia. (E. P. B. 35 and 36, in the museum of the Army Medical Department.)

*Experiment* 12.—A piece of the shaft of a rabbit's tibia, weighing 1·5 grain, and a bit of the spongy extremity of the same bone, weighing one grain, were kept in the medullary cavity of another rabbit's tibia for twenty-five days. The weights were marked on these portions of bone with a black-lead pencil.

On being removed and dried, the first portion was found unchanged, and the second had increased one-tenth of a grain in weight, probably from matter which had not been dissipated in drying. The pencil marks were not obliterated.

There was much inflammation of the limb, and pus with vascular lymph surrounded the adventitious portions of bone. (E. P. B, 48 and 49, in the museum of the Army Medical Department.)

*Experiment* 15.—A bit of the shaft of a rabbit's tibia, weighing 2·2 grains, was introduced into the tube of another rabbit's tibia, and kept there seven weeks. The wound healed in the course of a few days.

The adventitious bone weighed 2·37 grains, and it was firmly imbedded in the medullary canal. The increase of weight was accounted for by two well-defined specks of new osseous matter deposited on its surface; and these deposits were removed and analysed by Dr. Davy, who found their composition to be that of true bone. (E. P. B. 57 and 58, in the museum of the Army Medical Department.)

*Experiment* 19.—A splint of a man's bone was introduced into the medullary canal of a rabbit's tibia. The animal became healthy and playful after the operation, and was kept as a pet in the house, for upwards of fifteen months, until it died. The inclosed bone was found to have suffered no change; it was separated from the tibia, which was somewhat thickened, by boiling. (C. 58, in Mr. Liston's collection.)

The foregoing experiments are certainly interesting, and must tend to breed extreme scepticism with regard to the absorption of dead bone. Mr. Gulliver remarks, and fairly enough, that:—

"These experiments are selected from a great number which I have made, all tending to the same conclusion. They have not been sufficiently varied and extensive to admit of being adduced as peremptory proof of the impossibility of the absorption of dead bone, in opposition to the incontestable power of the absorbents in the removal of inorganic particles from the living body, but I conceive that it is now fully established, with how much difficulty dead bone is subject to absorption, and that whatever may be the agency of this process in the removal of living parts, it can no longer be regarded as the means by which the sequestrum disappears in cases of necrosis" 18.

There is a point to which Mr. Gulliver directs attention, and which, undoubtedly deserves it—the deposition of new bone upon the old, and their adhesion or consolidation.

"It appears to me to be a very interesting fact, that a tissue which has been long dead should possess the power of attracting, as it were, particles similar to itself from the blood. To complete the resemblance to assimilation, we have only to suppose the dead matter to be porous, and the new particles attracted to its interstices." 19.

Unless our ideas on the subject of the connexion of living and dead parts are erroneous, we must suppose that some vitalization of the included bone took place, in order to enable the new bone to adhere to it. If such adhesion could occur, why should not absorption? The latter appears to imply less vital force and exertion, because the absorbed part may be passive, as the aliment received into the system is. But leaving this question, we beg to call our readers' attention to the entire memoir, and the actual experiments.

### VII. ON THE PROPORTIONS OF ANIMAL AND EARTHY MATTER IN THE DIFFERENT BONES OF THE HUMAN BODY. By G. O. REES, M.D. F.G.S.

The object of Dr. Rees has been to determine the cause of the great discordance in the results of chemists who have occupied themselves in determining the proportions of the earthy and animal matter contained in human bone. He thought that different bones might possibly possess different quantities of each. The result of analysis has proved this supposition correct. It seems tolerably certain that the differences in the results of chemists may be assigned to three different causes, viz.

"1st. The employment of different bones for analysis; nearly every bone having a proportion of earthy and animal matter peculiar to itself.

2nd. The bones used for examination being differently prepared, and containing more or less of fat, which will be estimated in the analysis as animal matter of bone, whereas it is merely an infiltration into its structure.

3d. The loss of different quantities of carbonic acid during decarbonization, owing to its conversion into carbonic oxide gas, which escapes at a low heat from carbonate of lime when carbonaceous matter is present. A portion of carbonic acid must almost necessarily be lost by bone-ash during incineration." 408.

The experiments of Dr. Rees were made on bones from the same adult.

They were similarly prepared, quite dry, and free from fat, periosteum, and cartilage. After the decarbonization of each specimen, he took the precaution of supplying the loss of carbonic acid which it had experienced, by moistening the result with a solution of sesqui-carbonate of ammonia, and then carefully applying heat to low redness.

The following were the results of analysis:—

| | Earthy matter. | Animal matter. |
|---|---|---|
| * Femur | 62·49 | 37·51 |
| * Tibia | 60·01 | 39·99 |
| * Fibula | 60·02 | 39·98 |
| * Humerus | 63·02 | 36·98 |
| * Ulna | 60·50 | 39·50 |
| * Radius | 60·51 | 39·49 |
| Temporal bone† | 63·50 | 36·50 |
| Vertebra‡ | 57·42 | 42·58 |
| Rib§ | 57·49 | 42·51 |
| Clavicle | 57·52 | 42·48 |
| Ilium ‖ | 58·79 | 41·21 |
| Scapula ¶ | 54·51 | 45·49 |
| Sternum | 56·00 | 44·00 |
| Metatarsal bone of great toe | 56·53 | 43·47 |

The following conclusions seem to flow naturally from the foregoing analyses:—

1st. The long bones of the extremities contain more earthy matter than those of the trunk.

2d. The bones of the upper extremity contain somewhat more earthy matter than the corresponding bones of the lower extremity; thus the humerus has more than the femur, and the radius and ulna more than the tibia and fibula: this difference is, however, small, being about one-half per cent.

3d. The humerus contains more earthy matter than the radius and ulna; and the femur more than the tibia and fibula.

4th. The tibia and fibula contain, as nearly as possible, the same proportions of animal and earthy matter, and the radius and ulna may also be considered alike in constitution.

5th. The vertebra, rib, and clavicle are nearly identical as regards the proportion of earthy matter; the ilium containing somewhat more of earths, the scapula and sternum somewhat less; the sternum containing more earthy matter than the scapula.

6th. The bones of the head contain considerably more earthy matter than the bones of the trunk, as observed by Dr. J. Davy; but the humerus and other long bones are very nearly as rich in earths.

7th. The metatarsal bones may probably be ranked with those of the trunk in proportional constitution.

---

* Solid parts of the shafts were used for experiment.

† Hard squamous portion.

‡ Arch of dorsal.

§ Solid external crust.

‖ Near the crest.

¶ Coracoid process.

To determine the correctness, or otherwise, of the supposition that the more cellular bones and the cancellated structure contain an increased proportion of animal matter, Dr. Rees made the following analyses:

| | Earthy matter. | Animal matter. |
|---|---|---|
| Cancellated structure from the head of the femur | 60·81 | 39·19 |
| Cancellated structure from the body of a rib | 53·12 | 46·88 |
| Solid structure of the same rib | 57·77 | 42·23 |

The cancellated structure in the rib does therefore contain less earthy matter than its compact tissue.

Dr. Rees was curious to ascertain whether the same law of relative proportion held good in the fœtal and adult skeleton. He procured several bones of a fœtus, full grown within a few days, and analysed them, with the following result:—

| | Earthy matter. | Animal matter. |
|---|---|---|
| Femur | 57·51 | 42·49 |
| Tibia | 56·52 | 43·48 |
| Fibula | 56·00 | 44·00 |
| Humerus | 58·08 | 41·92 |
| Radius | 56·50 | 43·50 |
| Ulna | 57·49 | 42·51 |
| Clavicle | 56·75 | 43·25 |
| Ilium | 58·50 | 41·50 |
| Scapula | 56·60 | 43·40 |
| Rib | 57·35 | 42·65 |
| Parietal bone | 55·90 | 44·10 |

Thus, in fœtal as in adult bones, those of the upper extremity contain somewhat more earthy matter than the corresponding bones of the lower extremity.

The humerus contains more earthy matter than the radius or ulna, and the femur more than the tibia or fibula.

The ilium contains somewhat more, and the scapula somewhat less earthy matter than the clavicle or rib.

" The great difference," concludes Dr. Rees, "observable in the proportional constitution of the adult and fœtal bones, consists in the fact, that the long bones and the bones of the head in the fœtus, do not contain the excess of earthy matter which we observe in those of the adult. Thus the humerus of the fœtus, which is the richest in earthy matter of the long bones, contains 58·08 per cent. of earths, while the ilium of the same subject is found to contain 58·5 per cent. of earthy matter. The parietal bone, which was examined as the type of the cranial bones, gave a proportion of earthy matter less than that of any bone that I have examined. The results of the analyses of the bones of the trunk in the fœtal skeleton shew that they contain animal and earthy matter in the proportions of the adult; and therefore that the difference of compactness observed between them must be the result of mechanical arrangement rather than a difference in the proportion of earthy and animal matter. There is little doubt that the general conclusion that fœtal bones are deficient in earthy material, has been derived from comparative experiments made on the long bones of the extremities, where such deficiency certainly exists. I subjoin for comparison the per centage of earthy matter contained in some of the bones of the fœtus and adult.

| | Fœtus. | Adult. |
|---|---|---|
| Rib .. .. .. | 57·35 per cent. of earths | 57·49 per cent. |
| Ilium . .. .. | 58·50 .. .. .. .. .. | 58·79 |
| Scapula .. .. | 56·60 .. .. .. .. .. | 54·51 |
| Clavicle .. .. | 56·75 .. .. .. .. .. | 57·52 |

From this comparison, it appears that the bones of the trunk in the fœtal skeleton are as rich in the proportion of earthy matter as those of the adult; at least the difference is too small to be material. The deficiency of earthy matter in the bones of the fœtal extremities is simply explicable on the fact that such an excess of earths as appears necessary to very great strength of bone is not needed at birth, and therefore only appears in after-life." 413.

A very interesting paper.

We turn to the pathological memoirs.

VIII. Facts and Inferences relative to the Condition of the Vital Organs and Viscera in general, as to their Nutrition in certain Chronic Diseases. By John Clendinning, M.D. Physician to the St. Marylebone Infirmary, &c.

This is an attempt to apply the numeral or statistical calculus to the phenomena of disease. Our readers must be aware that we have warmly encouraged upon all occasions the employment of this exact method in medicine. It is calculated to correct some errors, establish upon surer grounds some truths, and to put us in possession of some of those wide generalizations which become a fixed basis for special inquiries.

But we have observed that a few enthusiastic persons, in and out of the profession, have anticipated the most extravagant results from statistical calculations. They have promised us a sort of millennium. We are to learn from them how many days in the year we shall be sick, how many years in a century we shall live, what will be the duration of any given disorder, how long the convalescence will last, what quantity of chicken-broth will be requisite, and how many stools a complaint will average. In short, we have heard the utmost possible nonsense uttered, and that nonsense the more ridiculous because it was clothed in the pomposity of numbers. These statists run mad have not known, or not remembered, that the disturbing circumstances of mode of life, atmospheric states, remedial treatment, even mental condition, are so great, that averages must be drawn from enormous numbers of individuals, and from the *very widest extremes*, to present even a semblance of truth as an *average*. The worth of this in its individual application afterwards may be guessed.

The object of Dr. Clendinning's paper is no such fool's chase. It applies itself to the solution of this question:—What are the modifications impressed on the nutritive functions in the viscera in certain chronic diseases? Does (ex. gr.) the defect of supply or excess of waste proceed in the same manner amongst the external and internal parts in phthisis? Does hypertrophy of the heart beget or indicate a general or partial tendency to hypertrophy? &c. &c.

"In answer to the questions just proposed, I proceed to offer some facts and observations. The facts I have to state consist principally of measurements by weight of nearly all the principal viscera in most cases, and of the person in many, of 249 subjects, of whose diseases and post-mortem appearances I am in possession of memoranda, taken, with a few exceptions, by myself. They are arranged in tabular form as follows.

Table 1 contains, in separate columns, the weight of the encephalon, heart, liver, kidneys, spleen, and pancreas of each of 31 males, dead of various known diseases, not *phthisis* or *morbus cordis*, between 21 and 60 years of age.

Table 2 contains like particulars of 44 females, dead under like conditions as to disease and age.

Table 3 contains like particulars of 37 males dead, not of *phthisis* or *morbus cordis*, at ages above 60 years of age.

Table 4 contains the weights of the hearts of 33 females of various ages above 60, and dead of various diseases exclusive of *phthisis* and *morbus cordis*.

Table 5 includes particulars arranged as above, of 27 males, dead of *phthisis* between 21 and 60 years of age.

Table 6 gives like particulars of 16 females, dead under similar conditions of age and disease.

Table 7 contains particulars, tabulated as before, of each of 41 males, dead of *morbus cordis*, between 21 and 60 years of age.

Table 8 contains for 20 females, dead of the same disease, and between 21 and 60, the like particulars.

In most of those tables the weights, in more or fewer instances, are given for the person and the stomach, and of nearly all cases the diseases are recorded.

With respect to the mode of obtaining the weights, it is proper to explain, that where the weight of the person is given, it comprehends the whole person, the viscera included. It was ascertained by means of a steelyard and must be accurate, although, I confess, that I often at first suspected important errors in the use of the instrument, owing to the instrumental weight differing so much, falling, in fact, so far short of the apparent weight, judging by the eye. The visceral weights are all avoirdupois, and were all ascertained by means of a balance, and are generally correct to within half a drachm. With regard to the results of weighing by the balance also I may mention, that I have often been surprised at the errors of my visual and manual estimates; errors like the former with the steelyard, generally much in defect and rarely in excess, and so difficult to avoid, that I confess I should feel little confidence in any estimate of the organized contents, or in other words, of the quantity or density of any viscus not tried by some test less fallacious than visual or manual estimate, except, of course, in case of very great and obvious excess or defect of quantity, which must necessarily be readily detected, although it could not, I believe, be measured with pretensions to accuracy without instrumental aid.

I may further mention, that before placing the viscera in the balance they were carefully separated from their appendages—the brain or encephalon and heart from their outer coverings—the liver, spleen, pancreas, kidneys, and stomach from fat, cellular substance, peritoneum, and other extrinsic parts; in fact, from all parts that were not included within the *tunica propria*, or that might in any way materially affect the result. The brain or encephalon, heart, and stomach were usually sliced, washed, &c., and the other viscera were generally similarly treated when congested or otherwise open to just suspicion." 39.

This quotation will give an idea of the mode of investigation adopted by Dr. Clendinning, a mode which those must know who have any intention of testing his results. His tables are too long for a periodical journal. We shall present a summary of their principal contents, chiefly in the words of Dr. Clendinning himself.

### 1. Phthisis.

If the data in question be true, we find that, in males between 21 and 60, and not labouring under consumption or disease of the heart, the average weight of the

| | | | |
|---|---|---|---|
| Brain...... will be .... | 46¼ oz. or | 20226 | grains. |
| Heart ................ | $9\frac{1}{10}$ ,, or | 3982 | grs. |
| Liver ................ | 53½ ,, or | 23408 | grs. |
| Kidneys ................ | 9⅕ ,, or | 4025 | grs. |
| Spleen................ | 5 ,, or | 2188 | grs. |
| Pancreas .............. | 2⅝ ,, or | 1148 | grs. |
| Stomach .............. | 5 ,, or | 2188 | grs. |
| Lungs................ | 46 ,, or | 20116 | grs. |
| Person .............. | 94½ lbs. or | 661500 | grs. |

According to the 5th Table, the weights in phthisis are, for the

| | | |
|---|---|---|
| Brain ................ | 46⅕ oz. instead of | 46¼ |
| Heart ................ | 9⅙ ............ | $9\frac{1}{10}$ |
| Liver ................ | 58½ ............ | 53½ |
| Kidneys .............. | 10¼ ............ | 9⅕ |
| Spleen ................ | 7½ ............ | 5 |
| Pancreas .............. | 3 ............ | 2⅝ |
| Stomach .............. | 5½ ............ | 5 |

The average weight of adult male phthisical subjects was under 94 lbs. avoirdupois, nearly 48 lbs. less than the average obtained for the healthy male of 40 years, by M. Quetelet. Thus a great disproportion obtains between the entire weight of the body, and that of the great viscera. The wasting of phthisis falls on the organs of locomotion, and on the external parts. The same holds in either sex. We see this from a comparison of the 2nd and 6th tables, containing, the former, particulars of 44 females, dead of various diseases, not phthisis or heart disease, the latter containing like particulars of 16 females dead of phthisis: the subjects of both Tables being between the ages of 21 and 60. The average weight of the whole subject, in the former female Table was 82lbs., that of the phthisical females was 66lbs., or more than a stone less than the former : yet, in most of the organs, the average weight was higher in the phthisical than in the other subjects.

Dr. Clendinning hangs on this pathological peg, some sanguine, we hope not fallacious anticipations.

"If fatal phthisis be, as waiving sympathetic functional disturbances, it would appear to be, essentially a local mischief; if with regard to its point of fatal attack, it be confined to the lungs, although indicating probably a constitutional propensity; if, amid all the waste of external non-vital organs and the vitiated nutrition of the pulmonary structures, the vital organs in general may, as they not unfrequently do, retain their normal structures and capacities; may we not hope that, in some future year, we shall learn to control the disintegrating processes, and correct the depraved nutrition, and heal the structnral lesions, and re-establish the functional capacities of the phthisical lung, with as much certainty and facility as we already experience in the cure of several diseases formerly very fatal, but now, in a large majority of cases, remediable by our still very imperfect therapeutical resources." 44.

### 2. MORBUS CORDIS.

*a.* In diseases of the heart, there is a remarkable superiority in bulk, or density, or both, of the important organs. On comparing Table 7, that of morbus cordis in males, with Table 1, representing the standard of health for males, we find that under every head, without exception, there is an excess in the former; the brain being in the morbus cordis Table about $\frac{1}{25}$th heavier than our standard—the heart being $\frac{2}{5}$ths heavier, the liver $\frac{1}{9}$th, the kidneys $\frac{1}{4}$th, the spleen $\frac{4}{5}$ths of an ounce heavier; the pancreas $\frac{1}{5}$th of an ounce; and the stomach also heavier than the standard.

On referring to the Table 8, that of morbus cordis in females, which is deduced from thirty observations, and comparing that Table with Table 2, the standard of health for females, results will be obtained almost the same as those from the male Tables. The brain of the female dead of morbus cordis was found heavier than the standard about $\frac{1}{14}$th, the heart nearly $\frac{1}{2}$ heavier, the liver $\frac{1}{12}$th, the kidneys $\frac{1}{8}$th, the spleen about $\frac{1}{4}$, the pancreas $\frac{1}{5}$th, the stomach more than $\frac{1}{4}$th, and the person nearly $\frac{1}{5}$th heavier. So that the female table fully confirms the male, and even with enlargement, as including the stomach and person, which are deficient in the first male table, or Table 1.

These positive facts are corroborated by the general observations, that have been made cursorily by pathological observers. Dr. Clendinning remarks, that, making every allowance for the firm, heavy, non-collapsing condition of the lung, in cardiac and asthmatic disease being due simply to œdema, still he has almost always found in chronic diseases of the heart, more especially in adult males, that plethora had existed and hypertrophy taken place in the branches of the air-tube; this is more particularly true of victims of heart disease that had survived the 40th year.

"Sometimes the hypertrophy, I have observed, is accompanied by dilatation, and constitutes Laennec's emphysema of the lungs. But sometimes, also, it is unattended by any expansion, or is accompanied by contraction, so that the air passage becomes nearly or wholly impervious; and this state of the bronchial twigs or branches seems to me to have been often mistaken for tuberculation, which it not a little resembles, and to have in consequence been called grey or miliary tuberculation. Into this, as it appears to me, erroneous view of the nature of a state of the lungs which is very common, and in adult males more common, I suspect, than true caseous tuberculation, have, in my judgment, fallen many of the first pathologists, amongst whom I would include the illustrious Laennec." 48.

But, Dr. Clendinning thinks, and perhaps with reason, that, not only is excessive supply of arterial fluids, by an enlarged left ventricle, a cause of much visceral disturbance and vitiation; but, there is a gradual alteration of the normal susceptibilities of the viscera, owing to which they become capable, without injury, of sustaining habitual venous congestion, and at length are enabled to resume, so to speak, their fœtal conditions, so far as to assimilate indifferently venous blood or the imperfectly renovated fluid, brought back from the unhealthy lungs by the arteries.

*b.* Passing over some speculative reasoning, as well as some correlative observations on this subject, we proceed to another—the comparative states of nutrition, as indicated by weight, at different ages.

"If we compare Table 1 with Table 3, we shall find that, according to my observations, advanced life is accompanied by shrinking or loss of substance in the case of every organ examined, with the single exception of the heart. The brain of males above 60 years of age appears from those tables to be about $\frac{1}{15}$th part lighter than that of adult males below 60 years of age; the liver about $\frac{1}{12}$th lighter; the kidneys $\frac{1}{8}$th lighter; the spleen $\frac{1}{2}$ lighter; the pancreas about $\frac{1}{3}$d lighter; and the person generally of course, though not noticed in both tables, much diminished in bulk and weight. The heart, however, instead of diminishing with the person and the viscera, generally seems to increase, and in the instances occurring to myself as above stated, to have exceeded on the average the normal standard by about $\frac{1}{12}$th part." 53.

Dr. Clendinning quotes some researches of Dr. Bigot's of Geneva. These confirm Dr. Clendinning's in one respect—the increase of the heart in advanced life; they oppose it in another; for Dr. Bigot maintains that the heart diminishes in phthisis. This latter observation agrees with our own. Every anatomist knows, that a phthisical heart is the best adapted for a preparation. It is wasted, thin, distensible from its flaccidity, and free from fat.

c. "I might also notice the preceding tables, Nos. 1, 2, 5, 6, 7, and 8, as illustrating the influence of sex, in modifying organic nutrition, as it appears from them, that whether in health or disease, there is in the male a greater development, arising of course from a more abundant nutrition, in the case of every organ examined, and that the excess on the side of the male, or, what is the same thing, the deficiency on the side of the female in disease of the heart, or phthisis, bears about the same proportion to the total weights of the encephalon of the other sex, that is found to exist in other diseases. This is an inference that might reasonably have been anticipated, but yet needed experimental proof. The inferior dimensions of the female person do not by any means necessarily imply corresponding visceral differences. This appears from several facts: Bigot, for example, found that the linear dimensions of the heart of 30 males of 60 French inches in height and under, exceeded those of the heart of 30 males of 60 inches in height and upwards; and he found the same rule nearly equally applicable to 18 females of 55 Paris inches stature and under, as compared with 34 females of 55 inches in height and upwards: and I have myself obtained similar results by a different method." 55.

Dr. Clendinning winds up his interesting paper with the following inferences. He does not, indeed he cannot, consider them conclusively established. But, no doubt, they approximate, more or less, to the truth.

"1. That the healthy adult male heart averages, for all ages under 60, nearly $8\frac{1}{2}$ ounces avoirdupois.

*Note.* This estimate agrees pretty well with the estimates of Senac, viz. 8 to 10 oz.; Bouillaud, 8 oz. and 3 grs. average; Cruveilhier, 7 to 8 oz. average; and Lobstein, 9 to 10 oz. average; considering that Senac and Lobstein made, as I recollect it, no distinction as to age or sex; while Bouillaud included in his estimate several hearts of subjects under 21 years of age, and Cruveilhier included subjects of various ages above 16 or 18, and of both sexes.

2. The healthy female adult heart averages nearly $7\frac{1}{2}$ ounces, or, more exactly, $7\frac{3}{8}$ ounces.

3. In phthisical subjects, the heart, in a large proportion of cases, (according to my observation,) weighs considerably more than in health.

4. The weight of the heart increases with years, up to the end of life, contrary to the law of nutrition of the viscera in general.

5. Hypertrophy of the heart generally, or of the left ventricle alone, predisposes not only to visceral and general plethora and hypertrophy, but also to acute and chronic inflammations in general, and especially to bronchitis, pneumonia, and pleurisy,—and the tendency it produces to disease of the bronchial ramifications in particular, and of the air vesicles, is such that cases of long standing are usually, if not invariably, complicated with chronic catarrh and emphysema of the lungs.

6. The average weight of the brain of the healthy adult male under 60 years of age is about 45·85 ounces: that of the healthy adult female under 60 about 41·25 ounces.

(*Note.* This is rather lower than the estimate of Dr. Sims, contained in his valuable paper in the 19th volume of the Transactions, which, for both sexes and all diseases, from 20 to 60 years of age, gives an average of about 45 ounces. But the estimate of Dr. Sims being founded on more than 100 observations of subjects between 20 and 60, is, in all probability, better entitled to confidence than mine, although taking no account of other difference than that of age.)

7. The weight and consequently nutrition of all the viscera exceed the normal standard in all cases of phthisis, in which the heart is increased in bulk or weight.

8. That in post-mortem inspections, more especially of cases of diseased heart, but also in other cases of which hypertrophy of any viscus might be supposed an element or complication, it is advisable, in addition to manual and visual examination and linear measurement, to employ other means, such as weighing, to ascertain accurately the state of nutrition and density of the viscera, and perhaps of the person, in order to avoid the risk of overlooking important deviations from the normal condition, not otherwise so readily and surely to be detected." 57.

### IX. ON INCREASED THICKNESS OF THE PARIETES OF ONE OF THE VENTRICLES OF THE HEART, WITH DIMINUTION OF ITS CAVITY. By GEORGE BUDD, M.B. F.R.S. Fellow of Caius College, Cambridge, and Physician to the Seamens' Hospital, Dreadnought.

M. Bertin, in 1811, applied the term "Concentric Hypertrophy" to unnatural thickness of the parietes of one of the ventricles of the heart, with diminution of its capacity. By this term, the condition in question has since been generally known, and it has been as generally considered a pathological state.

But M. Cruveilhier has lately taken a very different view of it. He says:—

"The facts, which I have had occasion to observe, do not allow me to admit concentric hypertrophy. the obliteration of the cavity, and the proportionably increased thickness of the parietes, appear to me the result of the mode of death. The hearts of all those whom I have had an opportunity of examining, who died by the executioner, have presented this double phenomenon in the highest degree; the parietes of the ventricle were in contact at all points. I have made the same observation with regard to other persons who died a violent death. The hearts concentrically hypertrophied, of the authors I have just quoted, (MM. Bertin and Bouillaud,) appear to me to be hearts, more or less hypertrophied, which death surprised in all their energy of contractility."*

* Dictionnaire de Méd. et de Chir. pratiques. Art. Hypertrophie.

To determine, so far as he can, which opinion is correct, has been the object of Dr. Budd's inquiries and of the present Paper. He refers to a considerable number of cases, some of which occurred to himself, while the great majority have been recorded by various authors.

1. The first eight cases are related somewhat in detail. It does not appear necessary to quote them, as the following brief summary of their leading features will present a sufficient account of them.

"Here then," says Dr. Budd, "we have eight cases, in which the appearances of concentric hypertrophy existed without complication of any considerable disease of the valves. In one of these only was any irregularity of the pulse noticed; in none was there dropsy; and in none, if we except Dr. Johnstone's case, in which there was a questionable dilatation of the right auricle, was there any dilatation of the right cavities. From this we may infer that the affection of the heart, in these cases, offered no considerable obstacle to the circulation through it. For when much obstacle exists, at least on the left side of the heart, there is generally intermittence or irregularity of the pulse, and almost invariably dilatation of the right cavities and dropsy." 305.

"By the absence, then, of these three conditions in the cases of concentric hypertrophy, we are justified in concluding that this affection, in the cases in which it has been observed uncomplicated with an obstruction at the valves, offered no obstacle to the circulation through the heart.

But how can we reconcile this with the smallness of the cavity in these cases? It is impossible to conceive that a left ventricle, which could scarcely hold an almond, should offer no obstacle to the circulation through the heart. Yet in this very case, the day before death, the pulse was quite natural in frequency, development, and rhythm, and we have the word of the accurate Laennec, that there was no symptom of disease of the heart.

In another very marked case, the pulse was noted as tolerably full and soft. None of these patients died of disease of the heart; and in all, the symptoms which could have led one to suspect cardiac disease were slight, and no other than those which indicate simple hypertrophy." 306.

Dr. Budd concludes that, in the cases related, they were hearts, more or less hypertrophied, which, to use the expression of Cruveilhier, death surprised in a state of contraction. Dr. Budd goes on to remark, that :—

Another inference from the preceding cases is, that enormous hypertrophy, unaccompanied by dilatation or by disease of the valves, does not produce any of the symptoms characteristic of an obstacle to the circulation through the heart. The true causes of these symptoms, when they exist in the heart, appear to be—

1. An increase in the volume of a cavity, relatively to the area of its discharging orifice, which renders necessary, as is evident from mechanical considerations, the exertion of greater force by the parietes to propel an equal quantity of blood with the same velocity.

2. Any obstruction from thickening or insufficiency of a valve.

3. A want of power in the parietes of a ventricle to empty the cavity, from deficiency of energy, as in cases of chlorosis, &c.

A further analysis of the cases seems to indicate some circumstances favourable to the appearance of concentric hypertrophy.

1. *Age.*—Six of the eight cases occurred in persons who had passed the meridian of life; four in persons who had reached the age of sixty or more; and, with one exception, the most marked cases occurred in the oldest persons. It is probable that the influence of age depends on its being favourable to hypertrophy. We may refer, upon this subject, to the researches of M. Bizot, and we may refer also, to the paper of Dr. Clendinning, which we have just analysed.

2. *Diseased Arteries.*—In six of these cases, there were considerable incrustations of the lining membrane of the arteries. This condition, by the resistance from friction which it offers to the course of the blood, is also a cause of hypertrophy.

4. *Emaciation.*—The subjects of four of the eight cases were noticed as being thin. The smallness of the quantity of blood may reasonably be supposed to have had some influence in producing the appearance in question. Dr. Budd observes that this supposition is countenanced by the frequency of "concentric hypertrophy" in the bodies of those who die of cholera.

5. *Mode of Death.*—In four at least of these cases death occurred from apoplexy.

II. Dr. Budd next proceeds to consider six cases, and to glance incidentally at several others, in which the concentric hypertrophy was accompanied by considerable valvular disease. The analysis is not a lengthened one, yet we must refer the reader who would examine it, to the paper itself. The following conclusions are all that we can quote.

"If we compare the cases in which the affection was unaccompanied by considerable obstacle from disease of the valves, with those in which such obstacle existed, we shall find that, in the first, there was no dropsy, no very evident signs of disease of the heart, and that neither of the patients died of a cardiac affection; that, of the others, there was dropsy in five cases; evident signs of the disease of the heart in all; and the disease of the heart the immediate cause of death in all. Now, the appearances of concentric hypertrophy were not more manifest in the second series than in the first.

If, then, the concentric hypertrophy observed in the second series was identical with that in the first, which it is fair to conclude for most of these cases, we must infer that the symptoms of disease of the heart, in the cases of the second series, did not result from the concentric hypertrophy, but from the valvular disease that accompanied it, and which was of itself, too, sufficient to account for such symptoms."* 312.

III. A remaining group of cases is to be disposed of—cases in which concentric hypertrophy was observed in conjunction with congenital malformation of the heart.

Dr. Budd relates five cases of concentric hypertrophy connected with congenital malformation. To use the summary of them given us by Dr. Budd, in all there was a congenital obstruction at the pulmonary orifice, and in most of them there was, certainly, concentric hypertrophy of the right ventricle. In the last of these cases, the circumstance of the child's dying at the age of thirteen days, proves that in it the concentric hypertrophy was also congenital; and as most of the other cases presented charac-

---

* "It is from not having distinguished the cases in which it occurs in conjunction with diseased valves that some physicians have considered pericarditis as a cause of hypertrophy."

ters similar, and differing only in degree, and as, in all, there was a malformation, evidently congenital, causing obstruction at the pulmonary orifice, we must, in Dr. Budd's opinion, conclude that, in these cases, the concentric hypertrophy was also congenital. He considers it proved that concentric hypertrophy of one of the ventricles of the heart, with obstruction at its discharging orifice, may exist as a congenital malformation, and that, in cases in which there is an extraordinary passage for the blood, through the foramen ovale or the ductus arteriosus, or by the communication between the ventricles, the natural thickness of the parietes may be increased five or six times, or even more; and that, generally, the right is the ventricle so affected.

The right auricle was greatly dilated in all the cases but one. All the patients, with one exception, died young. In the former categories, the affection was most frequently on the left side, and the patients were generally advanced in life. The paper terminates with the following recapitulation:—

"1. That similar appearances have been observed by M. Cruveilhier in the hearts of persons who died by the guillotine; and, by Mr. Jackson and others, in subjects whose death had been caused by cholera.

2. That in these cases the symptoms of cardiac disease were slight, and no other than those which indicate simple hypertrophy; and that there was no intermittence or irregularity of the pulse, no dilatation of the right cavities or dropsy; symptoms of obstacle to the circulation through the heart, which must have occurred had the cavity during life been so small as it appeared to be.

3. That, in two of the cases, the cavity was restored, by mechanical means, to its normal size; and that in none was there any obstacle behind it, by which its permanent diminution could be explained.

4. That the supposition of increased strength of the parietes with diminution of the cavity, and that, too, relatively to the area of its discharging orifice, is opposed by the mechanical considerations by which we account for the almost constant occurrence of hypertrophy in cases of dilatation.

II. In the six cases complicated by extensive valvular disease, the diminution of the cavity cannot be explained by the hypothesis of an obstacle behind it; and, in some of these cases, the existence of an obstacle before it renders it highly probable that this diminution was merely a passing condition of the ventricle: and, as the appearances of concentric hypertrophy were not more marked in these cases than in those of the former category, and as the symptoms of obstacle to the circulation, observed in these cases, were such as would result from the diseased valves alone, we cannot admit the existence of concentric hypertrophy in the category we are now considering.

III. Concentric hypertrophy of a ventricle, in a high degree, with obstruction at its discharging orifice, and an extraordinary passage for the blood, occasionally exists as a congenital malformation, and, in most cases, the right is the ventricle so affected.

IV. Hypertrophy of the heart, to whatever extent it exists, when it is exempt from dilatation of the cavities, and from disease of the valves, does not produce any of the symptoms of an obstacle to the circulation through the heart." 317.

Perhaps it may admit of doubt whether the second and the fourth propositions are satisfactorily established. Dr. Budd will, we think, admit that additional evidence and further investigations are desirable. But his paper will undoubtedly tend to disabuse the minds of medical men, of the vague ideas or the positive errors which occupied them on the subject of concentric hypertrophy. Dr. Budd has contributed some valuable observations to this volume of the Society's Transactions. He is a young physician of energy and promise.

The only remaining paper that we can notice is one by Mr. Thurnam, formerly apothecary to the Westminster Hospital. Those who have the pleasure of his acquaintance are aware of his industry and intelligence, and the paper we are about to examine is characteristic of both.

### X. ON ANEURYSMS OF THE HEART; WITH CASES. By JOHN THURNAM.

Partial dilatation, or aneurysm of the heart, attracted comparatively little attention till 1827, when the occurrence of several cases, nearly simultaneously, at Paris, gave rise to an important memoir from the pen of M. Breschet. That memoir contained the history of ten cases, and the inferences that appeared to be reasonably deduced from them. But other cases and more correct conclusions have, subsequently, been obtained, and the object of Mr. Thurnam is to present an account of what the existing amount of facts is capable of telling us.

"My attention," he says, "was first strongly directed to this disease, by the occurrence of a remarkable case of it in the Westminster Hospital, which will be the first narrated in this paper. I have since visited the different museums of this metropolis, and that at Fort Pitt, Chatham, and have thus had an opportunity of inspecting, at the least, twenty-five specimens of the lesion in a more or less advanced stage. Of these cases, I found that the greater proportion had not been published at all, and that many of the remainder had only been very imperfectly described in catalogues. Of the appearances of the disease in all these cases I have taken notes, and have endeavoured to obtain as much information respecting their history as possible; and in some instances, have succeeded in obtaining tolerably complete cases, which have been very obligingly confided to my disposal. The new cases with which I have in this way become acquainted are thirteen in number, and of these, eight I have detailed at length; of the others, the accounts are too defective for this purpose, but such particulars as I have been able to collect respecting them, as well as of others before described, I have availed myself of, and have arranged in an appendix to this paper, which contains every case of the affection with which I am acquainted. The materials thus collected are very considerable, amounting altogether to 84 cases, of which 58 are in the left ventricle. With such a number of facts before us, I cannot but conclude that a history of this disease may be formed, more complete than any we have hitherto possessed." 189.

Aneurysms of the heart, though most frequent in the left ventricle, have occurred in the left auricle, and, in a few rare instances, in the valves of the heart themselves. Mr. Thurnam treats of them in each of these three situations.

### A. ANEURYSM OF THE VENTRICLES.

So far as is known, the right ventricle is totally exempt from aneurysm. Putting aside the hypothetical explanations of the facts that have been offered, and some remarks of Mr. Thurnam's on the right and left hearts, we must mention the sense in which he employs the term *aneurysm*, in order that no misconception may exist with regard to his meaning. He understands by it:—

*An abnormal dilatation of a portion of the vascular system of red blood, either dependent upon, or necessarily connected with a morbid change in the tissues forming the walls of the dilated part.*

This definition will of course exclude not only all forms of dilatation of the right cavities of the heart and of the pulmonary artery, but also all general dilatations of the left cavities of the heart; different forms of which, either combined or uncombined with hypertrophy, have since the days of Baillie and Lancisi been generally known under the name of aneurysm. Mr. Thurnam points out the objections to the application of the term aneurysm to general dilatations of a cardiac cavity with or without hypertrophy—an application too generally made with the effect of producing inconvenience and confusion.

*a. Aneurysm of the Left Ventricle.* Mr. Thurnam relates, more or less circumstantially, the particulars of seven cases, and refers in an Appendix to fifty-one others. These details are too extensive for a Journal, and we must refer our readers to the Paper itself, if they are anxious to become acquainted with them. A summary of twenty-nine pages will enable us to obtain all the leading particulars, and the great generalizations. We shall condense, where condensation would be prudent, the analysis of Mr. Thurnam. Its numerical and statistical character renders compression almost impossible.

Lateral aneurysm, he says, of the left ventricle is met with under two principal forms. Thus it may be either unattended by any external deformity of the heart, and confined altogether to the ventricular walls; or it may present itself in the form of a tumor growing from the exterior of the organ, and in size varying from that of a nut to that of the heart itself. In sixty-seven aneurysms occurring in the fifty-eight cases, thirty-five were attended by tumor; in nineteen there was no tumor; and in the remaining thirteen, it is doubtful whether tumor existed or not; although, from the small size of the sacs in these latter cases, it is probable that the disease scarcely extended beyond the surface of the ventricle. There can scarcely be a doubt that, in its earlier stages at least, this lesion is far from unfrequent; and it may be observed, that it is in these stages that anatomical examination will be likely to throw light upon the mode of its formation.

*b.* The size of the sac varied from that of a nut to that of almost the healthy heart itself. In one case it had nearly projected externally. When the disease has been of some standing, and the sac has attained a certain size, it usually opens into the ventricle by a mouth, the diameter of which is narrow, relatively to that of the sac itself; and the lips of which, like those of old arterial aneurysms, are generally projecting, well-defined, and formed of a dense fibrous tissue.

"With respect to the tissues of the heart engaged in the formation of the aneurysmal sac, a careful analysis of the cases would seem to shew, that in fifteen, the sacs were formed by the muscular fibres and pericardium; in four, by the endocardium and pericardium only; in twenty-five, by all of the structures entering into the composition of the walls of the heart; whilst in twenty-three cases, the disease was either too far advanced, or the data are insufficient to enable us to assign them to their proper places. The aneurysmal sacs had in some cases undergone changes and transformations of different kinds; thus in two cases, they are stated to have assumed a steatomatous structure; in three, a cartilaginous one; which latter change, in six others, was combined with a more or less advanced calcareous or osseous degeneration." 219

*d*. In twenty-one cases, and probably in a still greater number, the sac had become strengthened by adhesion to the loose or fibrous layer of the pericardium; and in all these instances, the disease had advanced to the extent of producing tumor on the external surface of the heart. A very small tumor would appear adequate to the production of such adhesion. In a few cases there were only opacity and thickening, or shaggy false membranes on the surface of the sac.

*e*. "In six cases, in none of which had adhesion taken place between the aneurysmal portion of the heart and the pericardium, and in which the aneurysm scarcely, if at all, projected beyond the surface of the ventricle, a rupture of the sac had occurred, which had led to a fatal extravasation of blood, into the pericardium. In one case only, related by Sir Astley Cooper, does rupture appear to have occurred when there was the adhesion alluded to, and in this instance the left pleura was the seat of the hæmorrhage. In another instance, the tendinous centre of the diaphragm was adherent to the greater part of the sac, which was very large, and had a small supplementary pouch, with very thin walls engrafted upon it; and had this become the seat of a rupture, it must have led to extravasation into the peritoneum." 220.

*f*. In twenty-three cases, the sacs, chiefly those with constricted mouths, and of considerable size, contained a greater or less quantity of laminated coagula; seventeen, either apparently of less standing, or situated more in the direct channel of the blood, contained simple amorphous coagula; whilst nineteen appear to have been found empty after death; one contained a hollow globular coagulum; two, simple but ancient fibrinous ones.

*g*. No part of the ventricle is exempt from aneurysm, but the apex is its most frequent seat. Thus the sixty-seven aneurysms which occurred in the fifty-eight cases, omitting one case in which this is not mentioned, may, as regards situation, be thus distributed; at or near the apex of the ventricle, twenty-seven; in different points of the base, twenty-one; in intermediate portions of the lateral walls, fifteen; in the interventricular septum, three. In short, setting aside more minute considerations, the thinnest parts of the walls of the left ventricle, or the apex and the highest part of the base, are those which are much more frequently than any others the seat of the disease.

*h*. In general, or in fifty-two out of the fifty-eight cases, only one aneurysm existed in each; but in four cases two were met with in each; in one there were three; and in another four incipient aneurysms. In two instances, it is not improbable that two sacs which were originally distinct had coalesced, so as to form a single aneurysm; and in another case, three sacs appear to have united in this way.

*i*. "An important point in the history of lateral aneurysm of the heart, is that which relates to the other lesions of this organ, which are found to accompany it. To begin with the pericardium: in addition to the twenty cases already alluded to, in which there was adhesion to the surface of the aneurysmal tumour, we find that, in seven cases, there was general adhesion of this membrane to the surface of the heart; that in one, there was recent hæmorrhagic pericarditis; and that in three, there was dropsy of this cavity. In twelve cases, the endocardium is stated to have undergone different changes of structure; so as to have become either white, opaque, or thickened in the immediate neighbourhood of the sacs, or even more extensively; and in one case, there was a minute deposit of calcareous matter either in or beneath this membrane. The muscular substance of the ventricle was, in at the least nine cases, the seat of more or less extensive fibro-cellular degeneration, which was generally most marked around the sacs; in one case, there was a cartilaginous transformation; and in another, induration from a non-specified cause. In one instance, the walls of the ventricles are said to have been the seat of 'lardaceous tumours,' and in another, of extensively diffused suppuration. In numerous cases, there was a marked atrophy either of the fleshy columns which form the pillars of the mitral valve, or of the smaller ones, which constitute the net-work on the internal surface of the ventricle. The valves of the left cavities are stated to have been diseased in ten cases; in five of these the mitral valve was the seat of the lesion, and was constricted by cartilaginous or osseous deposit; in three, the aortic valves were diseased, and both these sets of valves were implicated in one example. In eight cases, the valves are reported to have been healthy; whilst, in the remainder, their condition is not mentioned." 224.

The majority of these changes are inflammatory, or allied to inflammation. From their variety, it appears that aneurysm of the heart cannot be regarded as exclusively dependent upon pathological changes in one only of the tissues entering into the composition of this organ.

*k.* In the fifty-seven cases of aneurysm, there are reported to have been general dilatation of the organ in three instances; dilatation with hypertrophy of all the cavities in three; dilatation with hypertrophy of the left ventricle in nine; simple dilatation of the left ventricle in four; and simple hypertrophy of the same cavity in two other cases.

The number of cases in which the heart is not stated to have been the subject of some lesion in addition to the aneurysm, does not exceed ten; and in three only is it positively stated to have been otherwise healthy.

### *Causes of the Disease.*

*a. Sex.*—Of forty cases, in which this is recorded, thirty occurred in males and ten in females.

*b. Age.*—The age of the patient is either stated or to be inferred with tolerable accuracy in thirty-five cases. The youngest patient appears to have been eighteen, and the oldest eighty-one years of age; and the whole of the cases may be arranged in decennial periods as follow:—

| | | |
|---|---|---|
| Under...... 21 | years of age; | 1 case. |
| From 21 to 30 | ................ | 9 cases. |
| 31 .. 40 | ................ | 4 .... |
| 41 .. 50 | ................ | 3 .... |
| 51 .. 60 | ................ | 6 .... |
| 61 .. 70 | ................ | 4 .... |
| 71 .. 80 | ................ | 7 .... |
| Above 80 | .................... | 1 case. |

*c. Occupation.*—As regards the occupation and mode of life, out of seventeen cases, all males, in which this is stated, it appears that there were one nobleman, one merchant, one tragedian, the celebrated Talma, two generals, one colonel, five private soldiers, one gondolier, one cabinet-maker, two tailors, and two victuallers. The inconsiderable number forbids positive inferences. But, as Mr. Thurnam remarks, it is singular that one half of the patients should have been soldiers.

*d.* Under the head of predisposing causes may be ranked intemperate habits in four cases, and rheumatic disease of the heart in two. Yet in other cases (*some* of six) the presence of universal adhesions of the pericardium renders it probable that rheumatism had existed. And this *may* explain the comparative frequency of the complaint in early life, a frequency not seen in other aneurysmal disorders.

The exciting cause of the disease would appear to have been external violence in the form of an injury of the chest in the case of the gondolier, a fit of violent anger in that of the nobleman, protracted mental anxiety in another instance, severe efforts on the stage in the character of Hamlet, in the case of Talma, and in a fifth instance, the retention of the breath during a military flogging.

*Pathological Summary.*

"From an examination then, of the anatomical details, as well as of the apparent causes of the disease, in reference to the determining of its nature; I come to the conclusion, that in twenty-two cases out of the fifty-eight, the aneurysm originated in a dilatation of all the structures entering into the composition of the walls of the heart; and in six in a solution of continuity of the lining membrane and in a stratum of muscular fibres, either as a consequence of ulceration, or, what is more probable, of rupture; whilst in the remaining thirty cases the disease was either too far advanced or the data given are insufficient to enable us to form a satisfactory opinion on this question.

I therefore conclude that this lesion, in by far the greater proportion of cases, is of the nature of *true aneurysm*; or that it has its origin in the dilatation of a portion of the walls of the heart, which has become less able to resist the distending force of the blood, during the ventricular systole, in consequence of organic changes in the tissues composing it. These changes may be confined to one of these tissues, as the endocardium; or they may involve that membrane and the muscular structure simultaneously; or, lastly, they may, I believe, originate in the pericardium, and be propagated from without inwards. In a great majority of instances, these changes would appear to have been the result of a more or less active antecedent inflammation.

I have, on one or two occasions, noticed an appearance on the internal surface of the left ventricle, which appears to me to have been the earliest stage of those pathological changes which terminate in the formation of true aneurysm. This consists in a more or less decided enlargement of one of the natural interspaces or depressions between the smaller fleshy columns. In one case which I have had a recent opportunity of examining, I met with a small cavity in the centre of the interventricular septum, which was capable of containing a small horse-bean. This cavity was evidently an enlargement of one of the natural sulci, which have been alluded to; it was traversed by the lining membrane of the heart, which in this particular spot was white and opaque, and it was only separated from the cavity of the right ventricle by a very thin stratum of muscular fibres, of a whitish appearance and dense fibrous texture.

Granting that the condition which has been now described, would, under certain circumstances, have led to the production of an aneurysm of the heart; or, in other words, that it constituted an aneurysm in its earliest stage, the observation must be regarded as important, and as fully confirming the view which has been advocated of the more usual mode of formation of true cardiac aneurysm." 230.

*a.* But *false aneurysm,* that is, aneurysm originating not in a partial dilatation, but in a partial rupture of the heart's parietes may, undoubtedly, take place also. Mr. Thurnam observes that, the examination of some cases and preparations would lead us to conclude that rupture of the heart, even when ultimately fatal, has not always been of momentary occurrence, but, on the contrary, has taken place very gradually, having commenced in the internal stratum of fibres, and only slowly spread to the external.

Mr. Thurnam does not deny the possibility of false aneurysm of the heart originating in ulceration, and in the discharge of the contents of abscesses and cysts into the cavity of the ventricle; but he is not satisfied of this having been the mode of production in any case with which he is acquainted.

*b. External mixed aneurysm*, or the supervention of a false upon a true aneurysm, does not occur in the pericardial portion of the aorta, in consequence of the absence of a distensible cellular coat to this portion of the artery, and hence lateral aneurysm in this situation usually proves fatal from rupture at an early period. For the same reason, mixed aneurysm does not occur in the heart; but as we have already seen, the aneurysmal sac usually soon gains an adhesion to the pericardium, by which means rupture is, in most cases, prevented.

c. True aneurysm, or that by dilatation, may either involve a limited point only, or the whole circumference of an artery; and in the latter case it constitutes a disease which has been variously named, 'preternatural dilatation,' 'cylindrical or fusiform aneurysm,' 'diffused true aneurysm,' and 'arteriectasy.' I am, I believe, the first to contend for the existence of an analogous form of aneurysmal dilatation in the heart; for, as I have observed when speaking particularly of the fourth case in this paper, the lesion in that instance would appear to merit the name of 'diffused true aneurysm of the heart.' Dr. Carswell and M. Cruveilhier have indeed each alluded to a case of extensively diffused true aneurysm of the heart, and the former has given a drawing of the disease, but in neither of these cases had the dilatation involved the entire circumference of the ventricle." 233.

*d.* It appears not improper, continues Mr. Thurnam, to designate by the name of *dissecting aneurysm of the heart*, that form of the disease, in which an aneurysm, as in Dr. Hope's cases, forms a canal under the lining membrane of the ventricle, which opens at some other point.

*e.* When the sac is formed solely by endocardium and pericardium, the case has been compared with that rare form of arterial aneurysm, in which the lining membrane of the vessel protrudes through a rupture in the middle tunic, constituting a lesion, which has been sometimes designated "aneurysma herniosum," and sometimes "internal mixed aneurysm."

*f.* In the case of an aneurysm seated in the interventricular septum becoming ruptured, so as to form a communication with a portion of the venous system,—the right ventricle—we should have a lesion produced altogether analogous to that which results from the wound of an artery and its accompanying vein, and to which the name of *spontaneous varicose aneurysm of the heart*, is perfectly applicable. Two of the cases detailed may *possibly* have been of such a character.

It must be owned, that the two last analogies, though ingenious, are far-fetched, and it may admit of question whether this pathological transcendentalism is really beneficial. But it is curious if not instructive. Mr. Thurnam terminates the summary by the remark:—

"We are then, I think, justified in asserting that, we find in the heart, with the exception of 'the external mixed aneurysm,' for the non-occurrence of which there is an anatomical cause, all the varieties of the disease which are met with in the arteries themselves; and that we cannot recognize the simple increase in the capacity of the cavities of this organ as constituting a lesion that ought to be spoken of as aneurysm." 235.

*Symptomatology and Diagnosis.* The existing information upon these heads is neither extensive nor precise. Probably, in its incipient state, aneurysm of the heart is not attended with any derangement of consequence in the functions of the organ. In two cases of the disease in an early stage, the absence of symptoms referrible to the heart is expressly stated.

*a.* "The mode of incursion of the disease differs remarkably in two classes of cases. Thus in three instances the attack was sudden, and attended with marked symptoms, analogous to those observed in cases of rupture of the heart, when this is not directly fatal; either in consequence of the rupture being incomplete, or from the opening being so small as to allow only of a very gradual effusion of blood into the pericardium. The most instructive of these cases is that of the nobleman, related by Galeati; who, after a violent fit of anger, was suddenly seized with severe præcordial pain, orthopnœa, agitation, fear of death, a disposition to syncope, and a vibratory, frequent but languid pulse." 235.

Probably, in such cases, the disease is of the nature of false aneurysm from rupture.

*b.* In the great majority of cases, however, the disease would seem to have had a very insidious origin, and to have been only very gradually announced by symptoms. Possibly these may be instances of true aneurysm.

*c.* In five cases, the symptoms of the disease are described generically as those of 'diseased heart.' In twenty-three cases in which the symptoms are given in detail, these, taken in the order of their frequency, were as follow; dyspnœa, in several instances amounting to the severest form of orthopnœa, in fifteen cases; præcordial pain of different characters, in one or two cases amounting merely to uneasiness, but in several others accompanied by a sense of weight, in fourteen; dropsy more or less extensive, in ten cases; palpitation in nine cases; anxiety, dread of death, or restlessness, in eight; and syncope, or a disposition to it, in three cases.

In addition to these symptoms others are also more rarely mentioned; such as cough, throbbing of the carotid arteries, pulsation of the jugular veins, livid or blue countenance, and hæmorrhage from the nose and lungs. The condition of the pulse is noted only in a few of the cases, and in seven of these it is stated to have been feeble, sometimes in an extreme degree.

*d.* In fourteen cases, there are notices of the duration of the symptoms. In one case, the patient died in ten days from the development of the lesion; in the other cases, the duration of the disease ranged from three or four months to fifteen years.

*e.* "In twenty-four cases, the mode of death is stated. In twelve of these in which it was very sudden, it arose, in three from syncope; in one from an unknown cause; and in eight from internal hæmorrhage. In six of these eight cases, the hæmorrhage was dependent upon a rupture of the aneurysmal sac into the pericardium; in one, upon a rupture of the sac into the left pleura; and in another upon a rupture of the substance of the ventricle itself, in the immediate neighbourhood of the sac. In four cases the patients appear to have died from an apoplectic or paralytic affection, and in one from epistaxis. In three cases the mode of death was the more ordinary one in heart affections, or that by apnœa (asphyxia,) and this, though not positively stated, was probably also the case in six other instances. In the following cases, six in number, as well as in the four apoplectic cases, death was evidently the result of complication with other diseases." 238.

*f.* Aneurysm of the heart being generally complicated with other lesions of the organ, the detection of pathognomic symptoms has been difficult, indeed impossible. The symptoms which have been observed may be broadly referred to such as would ensue from retardation of blood in the cavities of the organ, and consequent obstruction to the venous circulation—and to a variety of distressing sensations in the præcordial region, sensations also met with in angina pectoris and valvular disease. Mr. Thurnam indulges in some ingenious observations on these points.

*g.* The physical signs have either been imperfect, or imperfectly observed —in all probability, both. In three cases, the impulse of the left ventricle is stated to have been increased; in one, the action of the heart generally was forcible and tumultuous; and in two others, feeble and obscure. In four cases, a bellows or rasping sound was heard with the ventricular systole; and in a fifth case, a similar sound was heard to the left of the sternum. In one case, the character of the first sound was short, like that of the second.

Mr. Thurnam presents a letter from an able auscultator, Dr. C. I. B. Williams. It contains what we may term suggestions for a diagnosis, that is, a statement of what the signs would probably be. Dr. Williams confesses himself unable to speak from experience. We think it unnecessary to pursue the subject of diagnosis, enveloped as it is in doubt and obscurity. Practically it is of little consequence, for if there be any symptoms of cardiac disease at all, the general and particular treatment would be much the same as if we knew there was an aneurysm.

*Prognosis.* It would be unnecessary to dwell upon its *badness.*

*Treatment.* And it would be equally futile to discuss this.

### B. Aneurysm of the Auricles.

*a.* Mr. Thurnam has collected the particulars of eleven cases of aneurysm of the left auricle. The disease would appear to have been nearly uniformly of the diffused kind, and to have generally involved the entire sinus of the auricle.

*b.* The dilated walls of the cavity are often thickened, and the seat of fibro-cellular degeneration. The lining membrane is opaque, rough, and otherwise diseased, and in some cases even ossified, and is lined with fibrinous layers, very similar to those met with in arterial aneurysms. In all these cases, the lining membrane appears to have been continued into the interior of the dilated portion, which consequently merits the name of true aneurysm. Occasionally, the dilatation is confined to the auricular appendage, which becomes excessively distended with lamellated concretions.

In all the cases of this kind with which Mr. Thurnam is acquainted (they are nine), there was an extreme contraction of the mitral orifice, producing a difficult transmission of the blood from the left auricle. We have seen several cases of this kind. But in those the affection of the auricle was of the character of hypertrophy and dilatation, with or without inflammatory alterations of its internal membrane.

*c.* "In one case only with which I am acquainted, was the aneurysm of that circumscribed kind to which the term *lateral* or *sacculated* could be applied. In this case, there was a sac as big as a nut hanging over the base of the left ventricle, and containing dense fibrinous concretions and liquid blood, which communicated with the cavity of the auricle by a canaliculated pedicle an inch in length.

The case related by Penada, which has been cited by Dezeimeris and Ollivier as one of aneurysm of the left auricle, was, I incline to think, after an examination of it and of the accompanying engraving, merely an instance of ulceration." 246.

*d.* But Mr. Thurnam goes on to remark, after relating the particulars of a case of Mr. Langstaff's, it is not in cases of contracted mitral orifice alone, that the left auricle may become the seat of aneurysmal dilatation. A case communicated to Dionis is conclusive upon this point.

*Case.*—A soldier who had deserted, whilst in fear of pursuit, struck the left side of his chest forcibly against a tree, by which he was thrown from his horse. From this time he became the subject of severe pain, palpitation, and dyspnœa; and a large pulsating tumor gradually formed to the left of the sternum, which at last extended from the clavicle to the fifth rib. He died about a year after the accident. In addition to ununited fracture of the first four true ribs, empyema and abscesses in the lungs; the left auricle of the heart was found of immense size, giving rise to the external tumor. The pleura, or probably rather the pericardium, adhered closely to the enlarged auricle, the walls of which were an inch thick, of a dense cartilaginous structure internally, and full of grumous blood. The aorta, venæ cavæ, and pulmonary artery, and veins were healthy.

*e.* Mr. Thurnam refers to two or three cases, in which the right auricle was the seat of a lesion analogous to aneurysm of the left.

The most remarkable, he observes, of these appears to be the case of the captain of a vessel, also related by Dionis, who, after making powerful efforts to restrain a fit of violent anger, experienced dyspnœa and severe palpitation, with a pricking sensation about the heart. He died twelve years after the commencement of these symptoms, having previously suffered from anasarca, cold extremities, a great disposition to sleep, and his death having been preceded by profuse epistaxis. The right auricle was found enlarged to the size of the head of a newly-born infant, and contained a pint and a half of semi-coagulated blood. The dilated auricle was lined with a scaly osseous substance, like egg-shell, which kept it stretched. The pericardium was firmly adherent. Dionis attributes this immense dilatation to the distention and partial rupture of fibres, which occurred in consequence of the sudden ingress of blood into the auricle, during the violent fit of rage.

Nothing is to be said on the symptoms or the treatment of these affections.

### C. ANEURYSM OF THE VALVES OF THE HEART.

"The valves of the heart themselves, as was previously observed, are sometimes the seat of dilatations, which may properly enough be styled aneurysmal. Morand and Laennec have each published a case of this partial dilatation occurring in the mitral valve, in the form of a little pouch which projected into the left auricle. In both these cases, the aortic valves were the seat of extensive ossification, so that great obstruction to the passage of the blood into the aorta must have existed. Indeed, I think it not improbable that this circumstance determined the dilatations, which possibly occurred in the valves rather than in any other part of the walls of the ventricle, in consequence of their being weaker than usual, either from congenital or acquired defect." 251.

In a preparation at St. Thomas's Hospital, taken from a case of Mr. Posthlewaite's, of Chichester, the sac is seated in the large or right portion of the valve, and encroaches considerably upon the septum of the auricles, which is, as it were, pulled down, so as to form part of the sac; a circumstance which probably depended upon the tendinous ring, to which the margin of the mitral valve is attached, having likewise given way. The sac would contain a large walnut; and in the substance of the inter-auricular septum directly above it there is a distinct ecchymosis. The only aortic valve which remains in the preparation appears perfectly healthy.

The next case related by our author is one of aneurysmal dilatation of the tricuspid valve. The preparation is in the Royal College of Surgeons, in whose museum it was deposited by Mr. Lawrence Healy. The patient was a "blue boy."

The last case related is one of aneurysmal dilatation of one of the two aortic valves, the third being congenitally absent.

We have not space for the particulars of these cases, and we must bring this article to a conclusion, by presenting the terminal remarks of Mr. Thurnam on the subject.

"I shall only add," he says, "to this paper, a very few observations on the history of aneurysm of the valves of the heart, in addition to those which are appended to the particular cases. I should be inclined to believe that, generally speaking, aneurysms of the valves of the heart originate in a progressively advancing dilatation, unpreceded by rupture or ulceration; and that in fact they are true aneurysms.

It is, however, possible that the aneurysmal dilatation may have been preceded in some cases by the destruction of one of the laminæ of the endocardium forming the valve affected; and in such instances the lesion must of course be regarded as a false aneurysm.

The constantly recurring movements to which these portions of the heart's structure are subject, are obviously unfavourable to the formation of coagula in aneurysmal pouches in these situations; and indeed it does not appear that such coagula had formed in any of the cases.

It is perhaps scarcely necessary to point out that a lesion of this description must necessarily act in a more or less decided manner, as an obstruction to the flow of the blood out of the cavity immediately behind the valve which is the seat of such lesion; and that if the aneurysmal sac be perforated, either as the result of ulceration or rupture, a regurgitation of blood will be permitted from the cavity in front of the diseased valve. It will hence follow that the diagnosis of aneurysm of the valves will, for practical purposes, resolve itself into that of obstructive and regurgitant valvular disease; upon which any observations of mine would be superfluous after the information we have respecting it, in the different standard works upon diseases of the heart in general, and especially in those of Drs. Hope and Williams." 262.

It is unnecessary to say more in reference to this paper, than that it reflects the highest credit on its author.

We have now analysed, more or less copiously, all the Papers in the present volume of Transactions, with the exception of one by Dr. William Thomson, on Black Expectoration and the Deposition of Black Matters in the Lungs. This appears to be scarcely adapted for our pages, being, in a great measure, of a critical character. We noticed a former Part of this Paper, and as we are promised a future and concluding one, we shall reserve what we have to say until that makes its appearance.

